# Editorial: Biofabrication and synthetic biology for enhanced medicinal plant bioproduction

**DOI:** 10.3389/fpls.2026.1876560

**Published:** 2026-06-01

**Authors:** Zishan Ahmad, Mohammad Faisal, Marcos Edel Martinez-Montero

**Affiliations:** 1 State Key Laboratory of Tree Genetics and Breeding, Co Innovation Centre for Sustainable Forestry in Southern China, Bamboo Research Institute, School of Life Sciences, Nanjing Forestry University, Nanjing, Jiangsu, China; 2 Department of Botany and Microbiology, College of Science, King Saud University, Riyadh, Saudi Arabia; 3 University of Ciego de Ávila, Ciego de Ávila, Cuba

**Keywords:** medicinal plant engineering, plant nanobiotechnology, plant synthetic biology, plant tissue biofabrication, secondary metabolites

**Figure 1:**
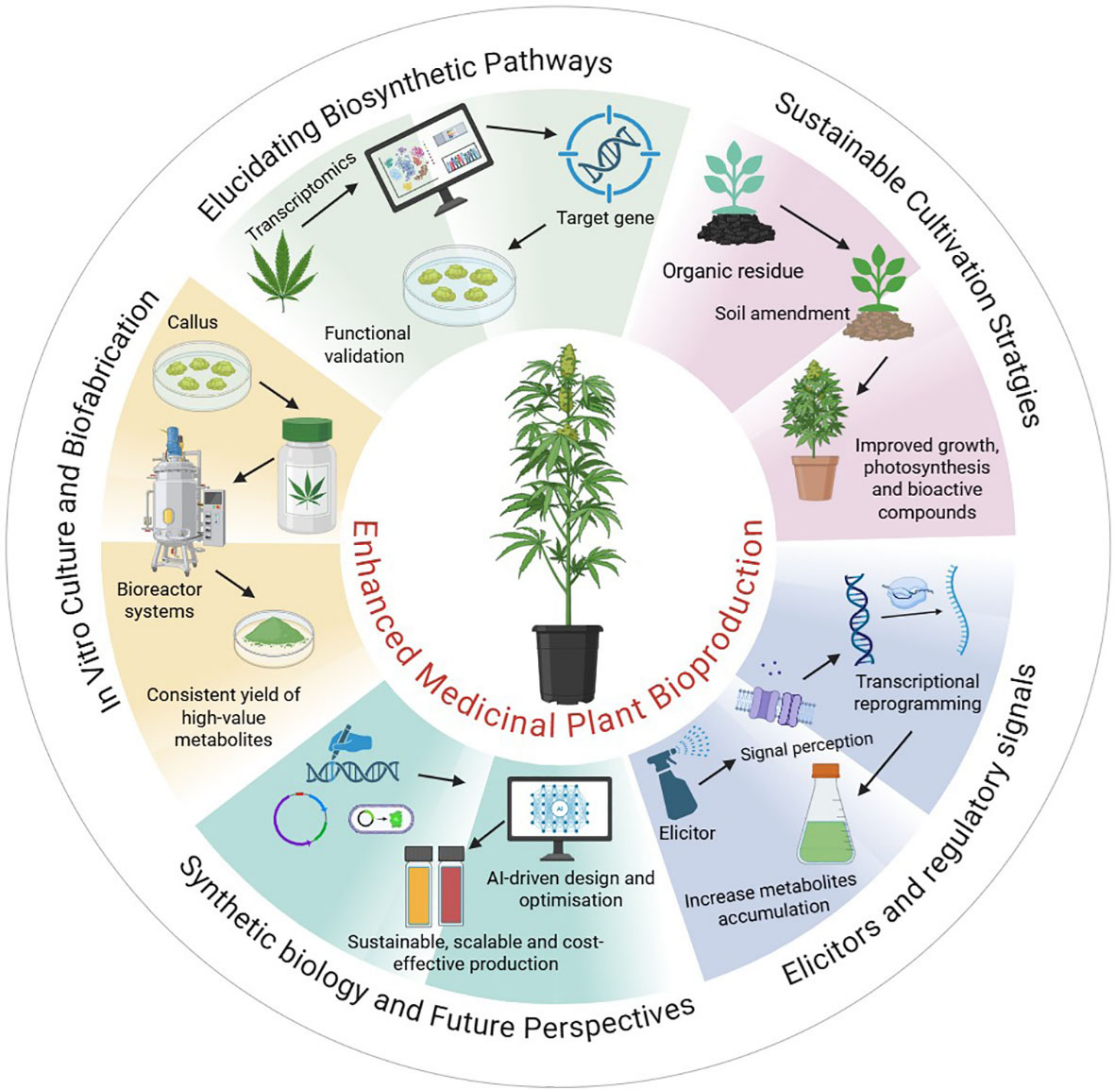
Biofabrication and synthetic biology for enhanced medicinal plant bioproduction – integrated multi omics, synthetic biology, innovative cultivation, and biofabrication strategies for sustainable production of high value phytochemicals. Created using https://www.biorender.com/.

Medicinal plants remain irreplaceable reservoirs of structurally complex, pharmacologically active specialized metabolites that underpin traditional and modern healthcare systems. However, transitioning these natural resources from traditional extraction to reliable industrial supply chains is severely constrained by overharvesting, environmental variability, and the inherent metabolic bottlenecks of slow growing species. To circumvent these limitations, the convergence of synthetic biology and biofabrication has emerged as a transformative paradigm. Synthetic biology provides the computational and genetic blueprints for precise pathway elucidation and metabolic flux rerouting, while biofabrication offers the engineered physical platforms from cell culture bioreactors to nano enabled cultivation systems required to execute these designs at scale (Seshadri et al., 2025; Firdaus et al., 2026) (**Figure 1**) (Seshadri et al., 2025; Firdaus et al., 2026). This molecular and physical synergy is further accelerated by pan genomic data, which elucidates how biosynthetic gene clusters bridge genetic variation with chemo diversity (Zhou and Liu, 2022). These results demonstrate how biosynthetic gene clusters and intricate genomic architectures connect genetic variation to chemodiversity. This Research Topic, *“Biofabrication and Synthetic Biology for Enhanced Medicinal Plant Bioproduction,”* brings together seven contributions that collectively highlight the integration of omics-driven discovery, metabolic engineering, innovative cultivation practices, and tissue culture technologies to enhance medicinal plant productivity and quality.

A foundational step in synthetic biology is the precise mapping of biosynthetic networks. Lian et al. and Pei et al. utilized SMRT sequencing to generate a full-length transcriptome of Rubia cordifolia, identifying 45,925 transcripts, 3,182 transcription factors, and 280 candidates involved in anthraquinone biosynthesis. By correlating isoform level expression with purpurin and mollugin accumulation, they defined an optimal harvest window (30 June – 15 October) – translating genomic data directly into agronomic utility. Similarly, Pei et al. coupled LC MS with transcriptomics in two Pinellia ternata varieties, identifying 63,106 unigenes and key alkaloid pathway genes (MAO, NCS I, NCS II, TyrAT). Overexpression in callus systems validated their roles in precursor channeling, demonstrating how systems level profiling transitions from gene discovery to functional validation. Collectively, these studies illustrate how multi-omics integration with functional validation can effectively link molecular mechanisms to pathway elucidation, and to useful applications in crop resource optimization and metabolic engineering.

Beyond molecular scales, this Research Topic highlights macro scale biofabrication and sustainable cultivation. Guan et al. applied 1 mmol/L methyl jasmonate (MeJA) to Blumea balsamifera, significantly upregulating MEP pathway genes and maximizing L borneol accumulation (3.346 mg·g⁻¹ FW in upper leaves at 120 h). This work underscores phytohormone mediated signaling as a powerful synthetic biology adjacent tool.

Beyond molecular scales, this Research Topic highlights macro scale biofabrication and sustainable cultivation. Liu et al. showed that soil amendment with *Polygonum cuspidatum* residues (2,500 kg/667 m²) enhanced endogenous phytohormones (IAA + 11.0 41.7%; ZR + 17.8 46.0%), upregulated *PcRS*, downregulated *PcPKS1*, and enhanced polydatin (+6.6 22.0%), emodin (+12.1 43.3%), and resveratrol (+17.8 69.3%). Similarly, Amir et al. demonstrated that phytofabricated gold nanoparticles (150 μM S AuNPs) improved chlorophyll levels, stomatal reopening via K^+^/Na^+^ modulation, and antioxidant activity in salt stressed spinach – establishing nano enabled priming as an indirect mechanism to preserve metabolic capacity for specialized metabolite synthesis under adverse conditions.

Controlled *in vitro* systems remain a core biofabrication platform. Bansal et al. performed untargeted metabolomics (GC MS, UPLC ESI QTOF MS) on Digitalis purpurea, revealing that tissue culture derived materials hyper accumulate >75 bioactive compounds (e.g., loliolide, stigmasterol, squalene) and cardiac glycosides (20,22 dihydrodigoxigenin, digoxigenin), with higher phenolics, flavonoids, and antioxidant activity than field grown plants. Cheng et al. comprehensively reviewed Lonicera japonica biotechnology, detailing how callus, hairy root, and protoplast systems can be dynamically optimized with elicitors (yeast polysaccharides, MeJA, UV B) for scalable chlorogenic acid production.

Collectively, the contributions of this Research Topic highlight several important themes that are influencing medicinal plant bioproduction. Firstly, complex biosynthetic pathways and potential genes for metabolic engineering are now analyzed based on the integration of multi-omics techniques, especially transcriptomics and metabolomics. Second, functional validation by genetic and biochemical methods is needed to validate gene function and to translate discoveries into useful applications. Third, sustainable and scalable production strategies, such as controlled culture systems and organic amendments, are growing in importance to meet global demand while lessening environmental impact. Finally, the convergence of a set of disciplines such as plant physiology, nanotechnology and synthetic biology is driving the innovation and the expansion of the application of biofabrication.

Despite significant progress, important challenges remain in fully reconstructing complex biosynthetic pathways, largely due to incomplete understanding of enzyme functions and regulatory networks. In addition, metabolic engineering techniques should also carefully balance the synthesis of secondary metabolites and growth to avoid unfavorable trade-offs. Overcoming these constraints will require more sophisticated computational techniques for pathway prediction, design and optimization, and more accurate genome editing tools (Chen et al., 2026). The convergence of artificial intelligence and machine learning with synthetic biology, particularly in combination with CRISPR/Cas-based genome engineering, is proposed as a future avenue to accelerate gene function annotation, pathway reconstruction and metabolic flux modeling. These advances combined should result in the more rational and predictive design of plant biosynthetic systems, enabling the precise bio-manufacturing of useful phytochemicals. The integration of molecular insights with engineering strategies can lead to the development of sustainable, scalable and effective platforms for high-value metabolite biosynthesis. This Research Topic provides a coherent summary of recent advances and new directions in biofabrication and synthetic biology for medicinal plant production.
